# Coexisting with large carnivores based on the Volterra principle

**DOI:** 10.1111/cobi.14448

**Published:** 2025-01-28

**Authors:** Mark S. Boyce, Cecile A. E. Carpentier, John D. C. Linnell

**Affiliations:** ^1^ Department of Biological Sciences University of Alberta Edmonton Alberta Canada; ^2^ Department of Forestry and Wildlife Management University of Inland Norway Koppang Norway; ^3^ Norwegian Institute for Nature Research Lillehammer Norway

**Keywords:** carnivores, coexistence, hunting, predation, predator–prey dynamics, Volterra principle, wolves, carnívoros, cacería, coexistencia, depredación, depredador‐presa, dinámicas, principio de Volterra

## Abstract

Coexistence with large carnivores represents one of the world's highest profile conservation challenges. Ecologists have identified ecological benefits derived from large carnivores (and large herbivores), yet livestock depredation, perceived competition for shared game, risks to pets and humans, and social conflicts often lead to demands for reduction of predator numbers from a range of stakeholder groups. Nearly 100 years ago, Vito Volterra predicted that increased mortality on both prey and predators results in increased abundance of prey and decreased abundance of predators. This principle appears to be robust and often consistent with the objectives of wildlife management. Although seldom recognized, and rarely tested in the field, the Volterra principle is a fundamental outcome of ecological theory with important implications for conservation.

## Abstract (es)

Coexistencia con carnívoros mayores basada en el principio de Volterra

**Resumen:**

La coexistencia con carnívoros mayores representa uno de los retos de conservación más importantes del mundo. Los ecólogos han identificado los beneficios ecológicos derivados de los carnívoros mayores (y de los grandes herbívoros), pero la depredación del ganado, la competencia percibida por la caza compartida, los riesgos para los animales domésticos y los humanos y los conflictos sociales a menudo conducen a demandas de reducción del número de depredadores por parte de una serie de grupos interesados. Hace casi 100 años, Vito Volterra predijo que el aumento de la mortalidad tanto de presas como de depredadores se traduce en un aumento de la abundancia de presas y una disminución de la abundancia de depredadores. Este principio parece sólido y a menudo coherente con los objetivos de la gestión de la fauna silvestre. Aunque rara vez se reconoce, y rara vez se pone a prueba sobre el terreno, el principio de Volterra es un resultado fundamental de la teoría ecológica con importantes implicaciones para la conservación.

## INTRODUCTION

One of the foundational principles of ecology emerged 100 years ago when Lotka (1925) and Volterra (1926) introduced a theory for predator–prey dynamics as a system of simultaneous differential equations. From these equations, the Italian mathematician Vito Volterra (1926) recognized the prediction that an increase in deaths for both predators and prey will result in a decrease in the number of predators and a nonintuitive increase in prey abundance (Abrams, 2009). Perhaps because the predictions were counterintuitive, few applications of Volterra's principle have been proposed. Our goal was to draw attention to the lasting value of the Volterra Principle by applying it to the high‐conflict conservation issue surrounding finding pathways to coexistence with recovering populations of large carnivores.

## VOLTERRA'S THIRD LAW

The world's first ecological model was a pair of simultaneous differential equations to represent the numerical interaction between populations of a prey (*N*_1_) and predator (*N*_2_):

(1) $$dN_{1}/dt=bN_{1}-dN_{1}N_{2}$$

and

(2) $$dN_{2}/dt=\beta N_{1}N_{2}-d_{2}N_{2},$$

where *b* is the exponential growth rate for a prey population without predation, δ is the prey killed by predators, *d*_2_ is the inherent death rate for the predator, and β is the production of young predators afforded by the consumption of prey.

The motivation for Volterra's model was the desire to understand observations made by his future son‐in‐law, fisheries biologist Umberto D'Ancona, that oscillations in abundance of Chondrichthyes (predators) and Osteichthyes (prey) in the Adriatic Sea occurred following World War I when fishing was stopped due to the war. Indeed, Volterra's law II is that his predator–prey model yields periodic oscillations in abundance, simply as a consequence of the interaction between predator and prey (Volterra, 1926).

Additional insight is obtained by examining equilibrium abundance. Setting both equations to zero shows equilibrium:

(3) $$0=bN_{1}-\delta N_{1}N_{2}\Rightarrow N_{2}*=b/\delta ,$$

where *N*_2_* is the equilibrium abundance of predators and δ is the prey killed by predators per unit time. Similarly, the equilibrium prey abundance, *N*_1_*, emerges:

(4) $$0=\beta N_{1}N_{2}-d_{2}N_{2}\Rightarrow N_{1}*=d_{2}/\beta$$

Volterra's (1926) insight was that if mortality were increased for both predator (*d*_2_) and prey (δ), the model predicts that at equilibrium one would observe an increase in prey and a decrease in predators, a result that he called the “third law.” For D'Ancona's fisheries example when postwar fishing resumed, harvests of Chondrichthyes (predators) decreased and Osteichthyes (prey) yields were higher under fishing that targeted both predators and prey. This continues even 80 years later (Legović, 2008).

A surprising result of the Volterra principle is that a prey population is predicted to increase when subjected to increased mortality. Indeed, the Volterra principle has been identified as one of the mechanisms that can lead to the “hydra effect” where the abundance of a population actually increases when harvested (Abrams, 2009).

**FIGURE 1:**
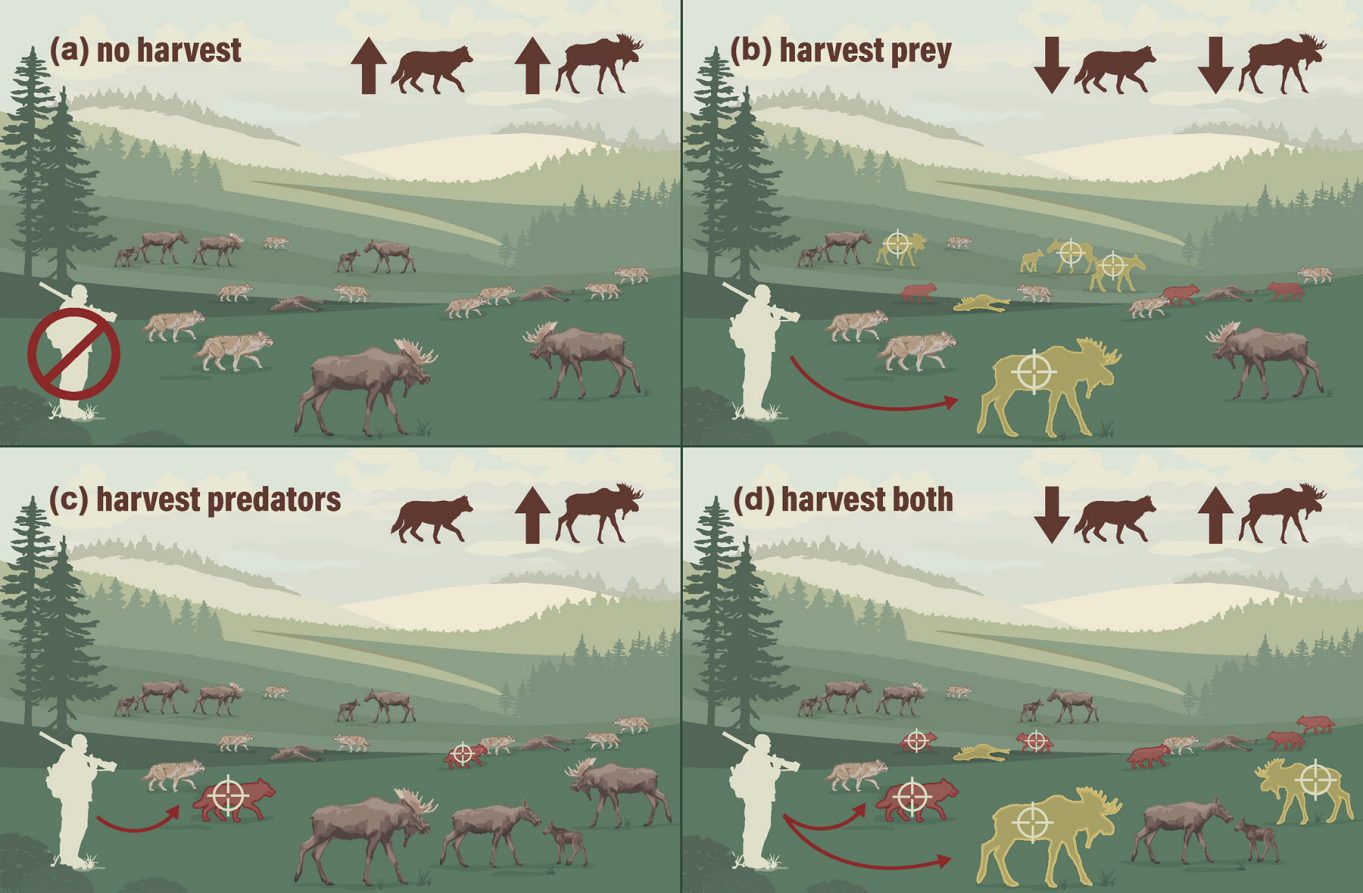
Alternative outcomes to (a) no harvest, (b) harvest of prey, (c) harvest of predators, and (d) harvest of prey and predators by humans in a predator–prey system, such as the Volterra model, applied to populations of moose and wolves.

Several other predictions emerge directly from this simple predator–prey model. As Volterra noted, increased protection of prey results in an increase in both predator and prey populations (i.e., bottom‐up increases). If hunting is imposed on the predator (*d*_2_) but not the prey, the model predicts an increase in prey but no particular response in predator abundance. Finally, increasing mortality on prey without predator hunting or control should decrease the abundance of predators (Carter et al., 2019). Clearly, predator abundance depends on the availability of prey (Karanth et al., 2004) (Figure 1).

## ROBUSTNESS OF THE VOLTERRA PRINCIPLE

The original model of Volterra is a simple 2‐species interactive model and as such does not include many attributes of predator–prey systems that are known to affect system behavior. Remarkably, however, adding complexity to the model does not change the overall predictions of the Volterra principle (Roughgarden, 1979). Specifically, when density dependence for prey, predator, or both is added, the Volterra principle holds. Likewise, the simplistic linear functional response can be relaxed by using an asymptotic (type II) or logistic (type III) functional response with no consequence to predictions of the Volterra principle (Roughgarden, 1979). Bellier et al. (2021) demonstrated the same outcome in models of a predator–prey system that incorporated different forms of stochasticity that affect both predator and prey and different harvest strategies. Predictions of the Volterra principle apply even when multiple predator species are competing for the same prey (Llibre & Xiao, 2014; Wang & Wu, 2020).

Mathematically, the Volterra principle also is robust to uncertainty in parameter estimates and model structure (Weisberg, 2006; Weisberg & Reisman, 2008). For example, increased harvests on both predator and prey result in decreased average predator abundance and increased average prey abundance (Volterra, 1926) even when the system is not at equilibrium. Likewise, the predictions of the Volterra principle persist under dynamic system properties, for example, complex bifurcated dynamics (Xiang et al., 2014) and multispecies systems (Abrams, 2009). Thus, even though one recognizes the simplicity of the original Volterra formulation, its fundamental structure captures key attributes of more complex predator–prey interactions that have potential ramifications for conservation. Even though the mathematical predictions of the Volterra principle are robust (Räz, 2017), for model predictions to have practical value, there is a clear need for empirical validation (Orzack & Sober, 1993). Unfortunately, relatively few demonstrations or experimental tests have been recognized. The original fisheries example (Volterra, 1926) has been reaffirmed (Legović, 2008). Applications of biocides for insect control have several examples that support predictions from the theory (Xiang et al., 2014). For example, postwar applications of DDT (dichloro‐diphenyl‐trichloroethane) increased cottony cushion scale insects (*Icerya purchasi*) but decreased abundance of ladybird beetles (MacArthur & Connell, 1966). Other examples might exist but have not been recognized as such.

## RECOVERY OF LARGE CARNIVORES AND THE SEARCH FOR COEXISTENCE

In contrast to the many global wildlife declines, large carnivore populations are recovering in many parts of the world, especially North America and Europe (Boyce, 2018; Chapron et al., 2014; Knopff et al., 2014; Tallian et al., 2021; Trump et al., 2022). Large carnivores can play an important role in ecosystem structure and function (Boyce, 2018; Terborgh & Estes, 2010), and appreciating this ecological role of large carnivores has been touted as the most important development in conservation science in recent decades (Ripple et al., 2014; Schmitz et al., 2023). However, the recovery of large carnivores also has met fierce resistance because the large spatial scales at which large carnivores operate inevitably mean that their conservation occurs in whole, or in part, in human‐dominated multiuse landscapes (Boitani & Linnell, 2015; Cretois et al., 2021; Ganz et al., 2024). Direct conflicts with large carnivores occur because of depredation on livestock (Morehouse & Boyce, 2011), impacts on threatened and endangered species (Hervieux et al., 2014; Lamb et al., 2024), pets (Bassi et al., 2021), competition with hunters for shared prey (Jonzén et al., 2013), and even attacks on humans (Linnell et al., 2021).

In light of these challenges, there are currently many debates about how conflicts can be managed and how sustainable coexistence can be achieved (Carter & Linnell, 2016, 2023). One of the emerging conflict dimensions concerns different visions of coexistence, most notably the appropriateness of the role of lethal management in the form of culling or hunting (Kaltenborn & Linnell, 2022; Lute et al., 2018). There is currently a strong social movement among some stakeholder groups toward a greater focus on nonlethal coexistence measures; however, lethal approaches are likely to remain a necessary part of an integrated coexistence toolkit (Allen et al., 2023). Essentially, we also believe that coexistence approaches will vary widely among regions, cultures, and geopolitical contexts. Against this background, we explored ecological principles that can guide discussions over the pros and cons of alternative coexistence approaches with a focus on those for wildlife management.

Although many conflicts can be prevented or mitigated by modification of human practices (Carter & Linnell, 2016), a common reaction to the above‐mentioned conflicts is to allow the killing of predators through either some form of regulated recreational harvest by hunters or more targeted operations by government agents (Treves, 2009). Another unofficial reaction to these conflicts includes illegal retributive killing of large carnivores by a small sector of society (Carter et al., 2017). The rationales for lethal control are diverse and variously involve goals of lowering predator density, selectively targeting problem individuals, or empowering local communities or adding value by embedding large carnivores in preexisting wildlife management (i.e., sustainable hunting) structures (Linnell et al., 2017; Redpath et al., 2017; Reynolds & Tapper, 1996).

Irrespective of justification, a widespread belief in many areas is that the price of coexistence in multiuse landscapes involves curtailing the natural trophic dynamics of predators, in much the same way wildlife management has done for overabundant large herbivores (Kuijper et al., 2016; Ordiz et al., 2013). However, predator control often is contentious among the public and conservation professionals (Bergstrom, 2017; Lennox et al, 2018; Lute et al., 2018) and sometimes has undesirable and unintended outcomes (Casanovas et al., 2012; Frey et al., 2022). While recognizing that much of the controversy surrounding the killing of predators concerns values and ethics associated with moral positions toward hunting in general and the killing of carnivores in particular (Kaltenborn & Linnell, 2022), there is a need for ecological science to inform public debate and decision‐making processes. Because many ecological systems are data poor, there is often a hope that science can provide heuristics, or simple rules of thumb or models, to guide decision‐making (Law et al., 2021).

To anticipate the consequences of actively managing the recovery of large carnivore populations, one can turn to basic ecological principles of predator–prey interactions, including the Volterra principle. We extended the model conceptually into a wildlife management situation in which humans, for example, act as a super predator, influencing mortality rates of both trophic levels. The prediction from the Volterra principle is that when both predator and prey are harvested by a superpredator, the outcome is an increase in the number of prey and a decrease in the number of predators (Ehrlich, 1986; Volterra, 1926). This prediction from theory is exactly the outcome that many wildlife managers desire, whereby the outcome is an increase in desirable ungulate prey (e.g., deer and moose [*Alces* sp.]), which provides game species and an opening for predator hunting opportunities for hunters and decreases populations of predators that frequently create human–wildlife conflicts but without involving exclusion or extirpation of the predator populations. Although this prediction from predator–prey theory has been known for nearly 100 years, it has not been recognized as a mechanism that can help justify multitrophic harvest‐based approaches to alleviate certain human–wildlife conflicts and promote sustainable forms of coexistence (Carter & Linnell, 2016, 2023).

We suggest that several applications can exist. In Alberta, Canada, populations of prey (elk [*Cervus canadensis*]) and predators (wolves [*Canis lupus*]) are hunted, and following wolf recovery >30 years ago, elk abundance and harvests have increased, consistent with the Volterra principle but counter to the initial expectations of wildlife managers (Trump et al., 2022). Wolves have been hunted and trapped at sustainable levels (Robichaud & Boyce, 2010); yet, surprisingly few livestock depredations were reported (Morehouse et al., 2018). The only exception was in the Rocky Mountains, where grizzly bears (*Ursus arctos*) have become highly effective predators of elk calves during spring (Berg et al., 2022; Griffin et al., 2011) and abundance and hunter harvest of elk have declined as grizzly bear populations increased and remain protected from hunter harvest (Trump et al., 2022). State agencies in the United States report the same patterns of elk hunter harvests in Idaho and Montana (M. Hurley & K. Proffitt, personal communication, 2023).

Circumstantial evidence also comes from counterfactuals. Since entering the European Union, legal constraints have led to a dramatic decrease in Eurasian lynx (*Lynx lynx*) harvests in Sweden, which has encouraged their recolonization. The result has been an increase in lynx numbers and a consequent decrease in the growth rate of their main prey, the roe deer (*Capreolus capreolus*), due to an increase in the predation pressure (Andrén & Liberg, 2015, 2024). In neighboring Norway, lynx numbers have been stabilized by hunting (Cusack et al., 2022), and trends in roe deer numbers have been relatively stable.

However, there are circumstances in which predictions of the Volterra principle fail. One cannot expect the predictions of the Volterra principle to function when the real‐world system deviates substantially from a modeled predator–prey system. Behavioral responses by prey to predators can obfuscate numerical responses; consider, for example, how fear of predators shapes prey behavior and habitat use (Ciuti et al., 2012; Santiago‐Avila et al., 2018). For Volterra principle predictions to apply would require a largely closed system such that dispersal and migration do not interfere with model predictions. Another example that might deviate from a real‐world predator–prey system is that of “apparent competition,” in which alternative prey results in elevated abundance of a common predator (Hervieux et al., 2014; Holt, 1977).

Another example differing from a simple predator–prey interaction includes populations of snowshoe hares (*Lepus americanus*) and Canadian lynx (*Lynx canadensis*) in North America. Long‐term studies and experiments show that this is not a simple predator–prey interaction but rather a 3‐way plant–herbivore–predator system (Krebs et al., 2001; Stenseth, 1995). Furthermore, dispersal by Canadian lynx is a key driver synchronizing population fluctuations over vast areas (Ranta et al., 1997), and spatial structure is not considered in the Volterra principle formulations.

## APPLICATIONS AND CAVEATS

Consider a wildlife management system in which both predator and prey are harvested (Peek et al., 2012) (e.g., wolves, moose, cougars [*Puma concolor*], mule deer [*Odocoileus hemionus*], Eurasian lynx, and roe deer [Andrén & Liberg, 2015, 2024]). The Volterra principle predicts that on average prey will be more abundant, thereby satisfying the interests of hunters who typically target cervids, whereas large carnivores, such as wolves and felids, will be at lower abundance, thereby reducing the risk of spillover conflicts when predators kill livestock, prey on pets, or threaten human safety—at least in situations where conflict is density dependent.

However, human–wildlife conflict can take many forms. In agricultural landscapes, abundant prey, such as ungulates, can themselves cause conflicts by damaging crops and forests or competing for food with livestock (Dickman, 2010). Even though reducing the density of large carnivores might contribute to reducing some conflicts (Comer, 2022; Garshelis et al., 2020; Mabille et al., 2015), the presence and behavior of large carnivores can lead to conflicts largely independent of their abundance levels predicted by the Volterra principle. For example, local socioeconomic and ecological context can be a major consideration in determining human–carnivore conflicts (Northrup et al. 2012; Haswell et al., 2016; Johnson & Wallach, 2016). Human practices (e.g., livestock protection, hunting, and recreational practices), the existence of attractants, and the degree of carnivore habituation are major factors behind many conflicts with large carnivores that might not be influenced by manipulating carnivore density (Comer, 2022; Morehouse & Boyce, 2017). However, the opportunity for removal of predators can alleviate some other conflicts simply because a landowner might believe that they have some degree of control over their situation (Anderson et al., 2024; Bruskotter & Wilson, 2014). In contrast, there is also the risk that lethal intervention can increase conflicts (Elbroch & Treves, 2023; Santiago‐Avila et al., 2018). For example, in British Columbia, Canada, human conflicts with cougars actually increased in a hunted population because hunters removed adult males that were replaced by multiple younger males more prone to being problem individuals (Teichman et al., 2016).

Large‐scale and systematic use of lethal killing of predators has been a common practice to alleviate livestock losses and enhance populations of game species for hunters (Berryman, 1972). Even in situations where predators are open for hunting or trapping, there is often a perception that more aggressive (larger magnitude) population reductions are required to achieve increased mortality on both predators and prey to achieve the results predicted from the Volterra principle (Hervieux et al., 2014). This is because of issues surrounding the sometimes‐limited efficacy of recreational hunters and trappers, especially their inability to respond to increased quotas (Bischof et al., 2012; Robichaud & Boyce, 2010).

For example, Alaska moose management (Boertje et al., 2010) suggests that predator control is necessary to push a wolf–moose system out of a predator pit (i.e., a predicted state with multiple equilibria). In practice, parameterizing a predator–prey model in which a predator pit emerges is virtually impossible, although presumably stochasticity can allow such things to happen (Clark et al., 2021). Indeed, predator–prey models are inherently dynamic especially when encumbered by seasonality and stochasticity (Boyce, 2000). However, predator control in Alaska has not achieved the desired increase in moose harvests (Miller et al., 2022) despite decades of predator control. Indeed, predator control has been reported to be ineffective in some cases because competing predators replace predation by a targeted species (Blythe & Boyce, 2020; Errington, 1946). Yet, these examples do not contradict predictions of the Volterra principle but show that a more complex system with coexisting predators also can yield the Volterra principle prediction (Abrams, 2009; Wang & Wu, 2020).

There is often concern that hunting of large carnivores will lead to their decline and extirpation. Certainly, the history of human exploitation of large carnivores reveals the potential impacts that humans can have when eradication was the objective or where humans were indifferent to the outcome. In contrast, we know of no examples from North America or Europe where regulated hunting of large carnivores (wolves, cougars, Eurasian lynx, brown/grizzly bears, and black bears) in modern adaptive management contexts has led to irreversible declines or unintended extirpation, although there is always room for improvement in terms of decision‐making processes and consideration of more subtle effects (e.g., Cusack et al., 2022; Frank et al., 2017).

Ecological processes, and conservation, are generally viewed as being highly contextual, with few rules or general principles that offer broad guidance. Nearly 100 years ago, Vito Volterra (1926) explained that the interaction between predator and prey yields a surprising result: that increasing mortality on both predator and prey results in an increase in abundance of prey and a decrease in abundance of predators. Wildlife managers appear to have overlooked the ramifications for conservation, whereby increased prey (e.g., deer, elk, and moose) is often desired by hunters and provides for lethal management of predators. Reductions in predator density could alleviate some conflicts for certain stakeholder groups and provide opportunities for predator hunting for others. Such sustainable management can afford multiple ecosystem services associated with wildlife harvests (Fromentin et al., 2022). Although ecological modeling simplifies nature to represent how systems work, Volterra's predictions appear to be remarkably robust. We hope to inspire further empirical investigations into how management can influence the complex ecological and social dynamics of present‐day ecosystems.
